# Hepatitis E virus infection in patients with acute non-A, non-B, non-C hepatitis in Central Brazil

**DOI:** 10.1590/0074-02760160256

**Published:** 2016-10-13

**Authors:** Nara Rubia de Freitas, Edna Braz Rocha de Santana, Ágabo Macedo da Costa e Silva, Sueli Meira da Silva, Sheila Araújo Teles, Noemi Rovaris Gardinali, Marcelo Alves Pinto, Regina Maria Bringel Martins

**Affiliations:** 1 Universidade Federal de Goiás, Instituto de Patologia Tropical e Saúde Pública, Goiânia, GO, Brasil; 2 Universidade Federal de Goiás, Faculdade de Enfermagem, Goiânia, GO, Brasil; 3 Fundação Oswaldo Cruz, Instituto Oswaldo Cruz, Rio de Janeiro, RJ, Brasil

**Keywords:** Hepatitis E virus, HEV, epidemiology, non-A, non-B, non-C hepatitis

## Abstract

Hepatitis E virus (HEV) infection has a worldwide distribution and represents an important cause of acute hepatitis. This study aims to investigate the occurrence of HEV infection and factors associated with this infection in patients with acute non-A, non-B, non-C hepatitis in Central Brazil. From April 2012 to October 2014, a cross-sectional study was conducted among 379 patients with acute non-A, non-B, non-C hepatitis in the City of Goiania, Central Brazil. Serum samples of all patients were tested for serological markers of HEV infection (anti-HEV IgM and IgG) by ELISA. Positive samples were confirmed using immunoblot test. Anti-HEV IgM and IgG positive samples were tested for HEV RNA. Of the 379 serum samples, one (0.3%) and 20 (5.3%) were positive for anti-HEV IgM and IgG, respectively. HEV RNA was not found in any sample positive for IgM and/or IgG anti-HEV. After multivariate analysis, low education level was independently associated with HEV seropositivity (p = 0.005), as well as living in rural area, with a borderline p-value (p = 0.056). In conclusion, HEV may be responsible for sporadic self-limited cases of acute hepatitis in Central Brazil.

Hepatitis E virus (HEV) infection has a worldwide distribution and represents a significant cause of acute hepatitis (Peréz-Gracia et al. 2015). Different epidemiological patterns are observed in developing and developed countries. HEV is mainly transmitted by the fecal-oral route in areas of lower socioeconomic status (Purdy & Khudyakov 2011) or in developed areas, by the consumption of animal meat exposed to HEV (Wenzel et al. 2011). In addition, recent studies have shown parenteral exposure as a possible route of HEV transmission (Hewitt et al. 2014, Ben-Ayed et al. 2015).

The signs and symptoms of hepatitis E, when present, are similar to other viral hepatitis. Therefore, patients with non-A, non-B, non-C hepatitis may represent a group at risk for HEV infection, which could lead to the need for a differential diagnosis. Serological diagnosis of HEV infection is performed through the detection of anti-HEV antibodies (IgM and IgG). Anti-HEV IgM antibodies are detectable in the early acute phase and disappear after four to six months, representing a suitable marker for the diagnosis of acute infection in immunocompetent individuals, while the presence of anti-HEV IgG marker indicates exposure to the virus (Khudyakov & Kamili 2011, Purdy & Khudyakov 2011, Krain et al. 2014). HEV RNA can be detected in the serum or stool of individuals during the acute phase of infection and its detection is especially useful in immunocompromised patients, especially for diagnosis and monitoring of chronic hepatitis E. In the acute infection, however, its value appears limited by the short period of viraemia (approximately two weeks) and release of viral particles in stool (approximately four weeks) (Krain et al. 2014).

In developed regions, studies conducted in patients with non-A, non-B, non-C hepatitis revealed anti-HEV IgM rates ranging from 0-11.4% (Buti et al. 1995, Echevarria et al. 2011) and anti-HEV IgG of 4.9-10.6% (Karetnyi et al. 1999, Inoue et al. 2009). Relative to HEV RNA, authors have detected rates ranging from 0-9.5% (Haagsman et al. 2007, Echevarria et al. 2011). In developing regions, most studies reported higher rates of anti-HEV IgM (20.2-60.5%), indicating a high prevalence of HEV acute infection (Abro et al. 2009, Blackard et al. 2009).

In Brazil, no routine testing is currently performed for HEV infection. The prevalence of anti-HEV IgG among patients with acute non-A, non-B, non-C hepatitis has been reported to range from 2.1% (3/146) to 29.0% (5/17) between 1992-1999 (Parana et al. 1997, Souto et al. 1997, Trinta et al. 2001, Lyra et al. 2005). In the latter study, anti-HEV IgM was detected in one out 12 patients (Lyra et al. 2005). From 2004-2008, one out 64 patients with acute non-A, non-B, non-C hepatitis was positive for IgM and IgG anti-HEV and also for HEV RNA (dos Santos et al. 2010). However, there is currently very little data on HEV infection among these patients in Brazil using new serological and molecular assays. This study aims to investigate the occurrence of HEV infection and factors associated with this infection in patients with acute non-A, non-B, non-C hepatitis in Central Brazil.

## MATERIALS AND METHODS

This cross-sectional study was carried out among patients of the Brazilian Public Health System (SUS) with acute non-A, non-B, non-C hepatitis, who were seen at a referral laboratory at the Federal University of Goiás, in the City of Goiânia (approximate population of 1,300,000), the capital of the state of Goiás, in Central Brazil. During the recruitment period (from April 2012 to October 2014), all patients were screened for viral hepatitis using the following criteria: acute hepatitis A by the presence of anti-HAV IgM, hepatitis B by the presence of HBsAg and anti-HBc IgM, and hepatitis C by detection of anti-HCV and HCV RNA. In addition, serum levels of alanine aminotransferase (ALT) and aspartate transaminase (AST) were determined. Those eligible to participate in this study were immunocompetent patients seronegative for hepatitis A (anti-HAV IgM), B (HBsAg and anti-HBc IgM) and C (anti-HCV and HCV RNA), had a clinical diagnosis suggestive of acute viral hepatitis (recent onset of signs and symptoms, such as jaundice, fever, fatigue and abdominal pain) and had abnormal transaminases (at least 2.5-fold higher than the upper normal limit). Viral hepatitis was diagnosed after the exclusion of other potential causes of liver injury, including alcohol abuse, treatment with potentially hepatotoxic drugs, biliary diseases and autoimmune hepatitis, as well as infections with cytomegalovirus and Epstein-Barr virus.

After written informed consent was obtained from all participants, they were interviewed using a standard questionnaire to collect data on sociodemographic and potential risk factors for HEV infection. Blood was collected (10 mL) from all participants and serum samples were stored at -20ºC for serological tests or -80ºC for molecular analysis. The protocol used for this study was approved by the Ethics Committee of the Federal University of Goiás (protocol 001/2010).

To evaluate HEV infection, all serum samples from the patients with acute non-A, non-B, non-C hepatitis were tested by ELISA for the presence of hepatitis E serological markers (anti-HEV IgM and IgG) (Recomwell HEV IgM and IgG, Mikrogen GmBH, Neuried, Germany). Positive samples were confirmed using immunoblot test (Recomline HEV IgG/IgM; Mikrogen GmbH). No commercial kit has ever been made available on the market in Brazil.

Anti-HEV IgM and IgG positive samples were further tested for the presence of HEV RNA. RNA was extracted and purified from 200 µL of serum using a Roche High Pure RNA Isolation kit (Roche Diagnostics GmbH Mannheim, Germany), following the manufacturer’s instructions. Real-time polymerase chain reaction (PCR) was performed for the detection (limit of four copies of HEV RNA per reaction) and quantification of the HEV RNA using TaqMan real-time PCR Technology, according to Jothikumar et al. (2006).

Prevalence and 95% confidence intervals (95% CI) were calculated. Factors associated with HEV infection (defined as positive for IgM and/or IgG) were initially assessed by univariate analysis. Categorical variables were compared using χ^2^ test or Fisher’s exact test. A p*-*value ≤ 0.10 was used to select variables that were included in a multivariate logistic regression model. A p*-*value of < 0.05 was defined as statistically significant. Data were analysed using the SPSS statistical package, version 20 (SPSS Inc., Chicago, USA).

## RESULTS

As shown in Table I, the mean age of the study group was 36.9 years (standard deviation = 17.2). Most patients were female (56.5%). Almost half of the study group (43.0%) was single, 59.1% were brown/*pardo* (mixed race), 53.3% had received more than nine years of formal education and 63.6% reported a family income above US$ 600 per month.

**TABLE I:** Sociodemographic characteristics of 379 patients with acute hepatitis non-A, non-B, non-C hepatitis in Central Brazil

Characteristics	(N)	(%)
Age (mean ± SD: 36.9 ± 17.2)		
≤ 35 years	194	51.2
> 35 years	185	48.8
Gender		
Female	214	56.5
Male	165	43.5
Marital status		
Single	163	43.0
Married	162	42.7
Divorced/widowed	54	14.3
Race/ethnicity		
White	95	25.1
Black	52	13.7
Brown/*pardo*	224	59.1
Yellow/Indigenous	8	2.1
Schooling (n = 370)		
< 5 years	39	10.5
5-9 years	134	36.2
> 9 years	197	53.3
Monthly income (n = 365)		
≤ US$ 600	133	36.4
> US$ 600	232	63.6

SD: standard deviation.

Of the 379 serum samples, only one was positive for anti-HEV IgM by ELISA and immunoblot. This sample was concurrently positive for anti-HEV IgG. Additionally, 21 other samples were also anti-HEV IgG positive by ELISA. After performing the immunoblot test, 20 samples were confirmed positive, revealing in an anti-HEV IgG positivity of 5.3% (Table II). HEV RNA was not found in any sample positive for IgM and/or IgG anti-HEV.

**TABLE II:** Seroprevalence of hepatitis E virus (HEV) markers among 379 patients with acute non-A, non-B, non-C hepatitis in Central Brazil

	Positive		
		
Markers	(N)	(%)	(95% CI)
IgM anti-HEV			
ELISA/Immunoblot	1	0.3	(0.0-1.7)
IgG anti-HEV			
ELISA	22	5.8	(3.8-8.8)
Immunoblot	20	5.3	(3.3-8.2)

CI: confidence interval.

Table III presents the factors associated with HEV infection. In the univariate analysis, age over 35 years, low education level, habit of bathing in the river and living in rural area were associated with anti-HEV positivity. After multivariate analysis, low education level was independently associated with HEV seropositivity (p = 0.005), and living in rural area was marginally associated (p = 0.056).

**TABLE III:** Factors associated with hepatitis E virus (HEV) among patients with acute non-A, non-B, non-C hepatitis in Central Brazil

Risk fator	HEV pos/total	(%)	OR (CI 95%)	p-value	Adjusted OR (95% CI)^*a*^	p-value
Age (years)						
≤ 35	4/194	2.1	1.0			
> 35	16/185	8.6	4.5 (1.5-13.7)	0.005	2.5 (0.7-8.6)	0.157
Gender						
Female	10/214	4.7	1.0			
Male	10/165	6.1	1.3 (0.5-3.2)	0.549	1.2 (0.5-3.2)	0.677
Schooling (n = 370)						
> 9 years	6/197	3.0	1.0			
5-9 years	6/134	4.5	1.5 (0.5-4.7)	0.496	1.2 (0.4-4.0)	0.717
< 5 years	8/39	20.5	8.2 (2.7-25.3)	0.001	5.4 (1.7-17.4)	0.005
Family income (n = 365)						
> US$ 600	12/232	5.2	1.0			
≤ US$ 600	8/133	6.0	1.2 (0.5-2.9)	0.734		
Habit of bathing in the river						
No	6/195	3.1	1.0			
Yes	14/184	7.6	2.6 (1.0-6.9)	0.049	2.0 (0.7-5.5)	0.189
Use of filtered water						
Yes	13/296	4.4	1.0			
No	7/83	8.4	2.0 (0.8-5.2)	0.153		
Animals at home						
No	5/118	4.2	1.0			
Yes	15/261	5.7	1.4 (0.5-3.9)	0.543		
Residence área						
Urban	3/177	1.7	1.0			
Rural	17/202	8.4	5.3 (1.5-18.5)	0.005	3.5 (1.0-12.9)	0.056
Pork meat consumption						
No	1/37	2.7	1.0			
Yes	19/342	5.6	2.1 (0.3-16.3)	0.707		
Bushmeat consumption						
No	5/130	3.8	1.0			
Yes	15/249	6.0	1.6 (0.6-4.5)	0.368		

*a*: adjusted for age, gender, schooling, habit of bathing in the river and residence area; OR: odds ratio; CI: confidence interval.

## DISCUSSION

In this study, only one sample was anti-HEV IgM positive, resulting in HEV acute infection rate of 0.3% (95% CI: 0.0-1.7). Similarly, another investigation conducted in Brazil in patients with non-A, non-B, non-C hepatitis revealed a low rate of acute hepatitis E (1.5%; 1/64) (dos Santos et al. 2010). On the other hand, anti-HEV IgM was detected in 27 out 552 (4.9%) samples from patients clinically suspected of hepatitis E analysed between the years 2006-2013 (Passos-Castilho et al. 2015). In other American and European countries, anti-HEV IgM rates ranged from 1.1-4.8% (Haagsman et al. 2007, Munné et al. 2011) except in groups with specific characteristics such as patients from outbreaks of acute viral hepatitis in Cuba (Lay et al. 2008) or hospitalised patients in Chile (Hurtado et al. 2005) and Italy (Romanò et al. 2011, Candido et al. 2012), where those rates were substantially higher (ranging from 20.6-40.3%).

The prevalence of anti-HEV IgG (5.3%; 95% CI: 3.3-8.2) found in this study was similar to that shown among blood donors in the same region (4.0%; 95% CI: 1.3-10.5) (da Silva et al. 2012). Relative to other Brazilian patients with non-A, non-B, non-C hepatitis, this prevalence was similar to that reported in the Southeast region (2.1%; 95% CI: 0.5-6.3) (Trinta et al. 2001), but it was lower than those observed in the Amazon and Northeast region (ranging from 12.5%; 95% CI: 2.2-37.2 to 29.0%; 95% CI: 11.4-55.9) (Parana et al. 1997, Souto et al. 1997). Recently, Passos-Castilho et al. (2015) have reported data of laboratory-based surveillance from 1998-2013 in a major clinical laboratory in São Paulo, in which a prevalence of 2.1% (95% CI: 1.5-2.8) for anti-HEV IgG was found. Nevertheless, it is important to emphasise that this was a retrospective study of 15 years of research (1998-2013). In addition, it is noteworthy that these studies have investigated population groups with different risk characteristics, and some of them have used a small sample. Moreover, none of these studies used confirmatory tests to estimate HEV prevalence.

Regarding the detection of anti-HEV IgG in patients with non-A, non-B, non-C hepatitis in other countries, the prevalence found in this study was similar to those observed in regions with low endemicity in the Americas, such as Argentina (3.0%; 95% CI: 1.3-6.1) (Munné et al. 2011), Colombia (7.5%; 95% CI: 5.1-11.0) (Peláez et al. 2014) and the US (4.9%; 95% CI: 2.5-9.1) (Karetnyi et al. 1999), as well as in Europe, including Hungary (7.2%; 95% CI: 4.5-11.2) (Haagsman et al. 2007), the Netherlands (5.8%; 95% CI: 3.6-9.2) (Herremans et al. 2007) and Spain (5.6%; 95% CI: 2.1-13.1) (Buti et al. 1995). On the other hand, among hospitalised patients with acute non-A, non-B, non-C hepatitis in Italy, anti-HEV IgG rates were higher (26.6%; 95% CI: 23.2-30.2 and 48.1%; 95% CI: 44.2-62.2) (Romanò et al. 2011, Candido et al. 2012).

Of the 22 anti-HEV IgG positive samples by ELISA, 20 were confirmed as positive by immunoblot, resulting in 91% of confirmation in this investigation. It was in accordance with other rates found in different population groups (67-97%) (Haagsman et al. 2007, Herremans et al. 2007, Vitral et al. 2014, Teshale et al. 2015). Relative to anti-HEV IgM detection, direct comparison is difficult because the only one positive sample by ELISA was also positive by immunoblot, but other studies revealed confirmation rates ranging from 20-75% (Haagsman et al. 2007, Herremans et al. 2007, Martins et al. 2014, Vitral et al. 2014). This demonstrates that the use of additional tests is important to eliminate false positives. As observed elsewhere (Lyra et al. 2005, Haagsman et al. 2007, Martins et al. 2014), the absence of HEV RNA can be explained by the short period of viraemia in cases of self-limited infection, which seems to be the profile of the study population.

In the multivariate analysis, low education level was associated with anti-HEV positivity, and living in a rural area, with a borderline p-value. Similarly, previous studies revealed higher rates of HEV infection in individuals with a low education level (Bortoliero et al. 2006, Martins et al. 2014). Additionally, other studies have reported that low educational level was statistically associated with HEV seropositivity (Junaid et al. 2014, Yoon et al. 2014) as well as living in rural area (Mansuy et al. 2011, Houcine et al. 2012). Most people who live in rural areas are farmers, suggesting potential exposure to HEV, either by contact with animals or by poor sanitary conditions (Labrique et al. 2009, Goumba et al. 2011, da Silva et al. 2012, Junaid et al. 2014).

This study had some limitations. Firstly, since this was a cross-sectional study, it was a single point examination without any follow-up. Secondly, the study population represents only patients with acute non-A, non-B, non-C hepatitis who were seen at a referral laboratory at the Federal University of Goiás (convenience sample) and, therefore, this population does not represent all patients with acute non-A, non-B, non-C hepatitis in our region. Thirdly, we have used an “in house” real-time PCR to detect HEV RNA in serum samples. In addition, the presence of HEV RNA was not examined in stool samples. Despite these limitations, this study provided a comprehensive investigation on HEV infection in a group of patients with acute non-A, non-B, non-C hepatitis in Central Brazil, indicating that HEV should be considered along with hepatitis A, B and C in the diagnosis of acute hepatitis.

In conclusion, our results indicate that HEV may be responsible for sporadic self-limited cases of acute hepatitis in Central Brazil. In addition, this study revealed a low prevalence of past HEV infection. HEV seropositivity was independently associated with low education level, and living in rural area (with a borderline p-value). These findings suggest that the HEV epidemiological profile observed is similar to that reported in regions with low endemicity.
